# PIKfyve-specific inhibitors restrict replication of multiple coronaviruses in vitro but not in a murine model of COVID-19

**DOI:** 10.1038/s42003-022-03766-2

**Published:** 2022-08-12

**Authors:** James Logue, Arup R. Chakraborty, Robert Johnson, Girija Goyal, Melissa Rodas, Louis J. Taylor, Lauren Baracco, Marisa E. McGrath, Robert Haupt, Brooke A. Furlong, Mercy Soong, Pranav Prabhala, Viktor Horvath, Kenneth E. Carlson, Stuart Weston, Donald E. Ingber, Melvin L. DePamphilis, Matthew B. Frieman

**Affiliations:** 1 Department of Microbiology and Immunology, University of Maryland, School of Medicine, 685 West Baltimore St, Baltimore, MD 21201 USA; 2 Center for Pathogen Research, University of Maryland, School of Medicine, 685 West Baltimore St, Baltimore, MD 21201 USA; 3 Division of Developmental Biology, National Institute of Child Health & Human Development, National Institutes of Health, Bethesda, MD 20892-2790 USA; 4 Wyss Institute for Biologically Inspired Engineering, Harvard University, Boston, MA 02115 USA; 5 Harvard John A. Paulson School of Engineering and Applied Sciences, Cambridge, MA 02139 USA; 6 Vascular Biology Program and Department of Surgery, Boston Children’s Hospital and Harvard Medical School, Boston, MA 02115 USA

**Keywords:** SARS-CoV-2, Virus-host interactions

## Abstract

The ongoing COVID-19 pandemic has claimed more than 6 million lives and continues to test the world economy and healthcare systems. To combat this pandemic, the biological research community has shifted efforts to the development of medical countermeasures, including vaccines and therapeutics. However, to date, the only small molecules approved for the treatment of COVID-19 in the United States are the nucleoside analogue Remdesivir and the protease inhibitor Paxlovid, though multiple compounds have received Emergency Use Authorization and many more are currently being tested in human efficacy trials. One such compound, Apilimod, is being considered as a COVID-19 therapeutic in a Phase II efficacy trial. However, at the time of writing, there are no published efficacy data in human trials or animal COVID-19 models. Here we show that, while Apilimod and other PIKfyve inhibitors have potent antiviral activity in various cell lines against multiple human coronaviruses, these compounds worsen disease in a COVID-19 murine model when given prophylactically or therapeutically.

## Abstract (web summary)

Compound-testing in a murine model for COVID-19 show that the prophylactic or therapeutic application of Apilimod and other PIKfyve inhibitors, the former being considered for human Phase II trials, worsens disease outcome with higher lung viral load and mortality.

## Introduction

Severe acute respiratory syndrome coronavirus 2 (SARS-CoV-2) is the etiologic agent of coronavirus disease 2019 (COVID-19), first reported in Wuhan City, Hubei Provence, China in early December 2019^1–3^. Since then, occurrence of COVID-19 has expanded to a worldwide pandemic, with over 400 million reported cases and over 6 million reported deaths at the time of writing^4,5^. In response, halting this pandemic has become a top priority for public health agencies and governments, spurring many research institutions to shift focus to SARS-CoV-2 as an all-in approach to solving this global problem. However, to date, very few small molecules therapeutics (Remdesivir, Molnupiravir, and Paxlovid) have attained approval or authorization from the Food and Drug Administration for the prevention or treatment of COVID-19^6,7^. Given the paucity of available medical countermeasures, continued research into compounds with the potential to treat this disease and their mechanisms of action is urgently needed.

SARS-CoV-2 is a positive-sense, single-stranded RNA virus that infects cells after binding of the viral spike glycoprotein to its target cell receptor, angiotensin-converting enzyme 2 (ACE2). Viral contents can then be released into the cytosol following spike cleavage by transmembrane protease serine 2 (TMPRSS2) on the cell surface or by cathepsin-mediated cleavage in endosomes^8–11^. Once inside the cell, SARS-CoV-2 generates a replication complex contained within double-membrane vesicles to avoid cellular detection, where subgenomic RNA (sgRNA) is transcribed and genomic RNA (gRNA) is replicated^12,13^. Proteins are then translated from sgRNA and translocate to the endoplasmic reticulum to facilitate the construction of new virions in the endoplasmic reticulum to Golgi intermediate compartment. Newly created virions are then secreted from the infected cell through the exocytosis pathway (reviewed in ref. ^13^). However, as SARS-CoV-2 is a newly identified virus, many of the virus lifecycle steps are posited from previous research on SARS-CoV, due to the similarity of the two viruses. Specific aspects of the SARS-CoV-2 lifecycle are still being actively researched.

As SARS-CoV-2 replication is reliant on host membrane synthesis and vesicular trafficking within cells, it may also be susceptible to therapeutic targeting of host trafficking machinery. An important enzyme involved in vesicle trafficking, phosphatidylinositol-3-phosphate 5-kinase type III (PIKfyve), has shown to be a promising in vitro therapeutic target for multiple diseases, including cancers, autoimmune diseases, and emerging viral diseases^14–20^. PIKfyve modifies a lipid involved in vesicle localization, phosphatidylinositol, by the addition of a 5′ phosphate, leading to the trafficking of multiple intracellular vesicles^21^. Though the PIKfyve inhibitor Apilimod has been shown to decrease SARS-CoV-2 infection in cell culture by blocking viral content release from endosomes and is currently being considered as a COVID-19 therapeutic in a Phase II clinical trial (listed as recruiting patients, NCT04446377), efficacy in COVID-19 animal models has yet to be reported^22,23^. In addition, other PIKfyve inhibitors, including the WX8-family of PIKfyve inhibitors, have yet to be studied^19^.

Here we describe the efficacy of multiple PIKfyve inhibitors against SARS-CoV-2 when administered pre- and post-infection in VeroE6 cells as well as in A549 cells overexpressing human ACE2 (A549/hACE2). We also describe the efficacy of these compounds in a murine model of COVID-19 to assess effects on lung infection and COVID-19 disease progression. Though these compounds showed nanomolar potency at disrupting SARS-CoV-2 replication in vitro, PIKfyve inhibitor administration prior to or following SARS-CoV-2 infection in a murine model of disease resulted in increases of both lung viral load and mortality as compared to vehicle-treated mice, likely due to a delayed but hyperactive immune response.

## Results

### PIKfyve inhibitors disrupt infection by multiple coronaviruses in vitro

To assess the efficacy of PIKfyve inhibitors against SARS-CoV-2 in vitro, VeroE6 or A549/hACE2 cells were pretreated with various concentration of PIKfyve inhibitors 2 h prior to infection with SARS-CoV-2 expressing green fluorescent protein (GFP). The percent of infected cells was then determined under these treatment conditions after nuclear staining and visualization using a Celigo cell imager (Nexclecom Inc). All inhibitors tested potently restricted SARS-CoV-2 replication in both cell lines, showing SARS-CoV-2 inhibition at nanomolar concentrations and minimal cytotoxicity (Table 1). We additionally tested the ability of Apilimod, WX8, and NDF to inhibit replication of hCoV-OC43, an endemic cold-causing coronavirus that infects humans. We found these PIKfyve inhibitors restricted hCoV-OC43 replication in human umbilical vein endothelial cells (HUVECs) as measured by infection-induced cell death, suggesting that PIKfyve inhibitors could be a potential pan-coronavirus therapeutic (Supplementary Fig. 1a).

**Table 1:** In vitro efficacy data for PIKfyve inhibitors against SARS-CoV-2 in VeroE6 and A549/hACE2 cells.

Compound	VeroE6 cells			A549/hACE2		
	IC50 (nM)	CC50 (nM)	SI	IC50 (nM)	CC50 (nM)	SI
Apilimod	10.05	>5000	>497.51	3.23	>5000	>1547.99
WX8	110.50	>5000	>45.25	22.62	>5000	>221.04
NDF	639.00	>5000	>7.82	9.39	>5000	>532.48
WWL	2110	>5000	>2.37	171.90	>5000	>29.09
XB6	1205	>5000	>4.15	191.00	>5000	>26.18
Remdesivir	546.5	>5000	>9.15	155.85	>5000	>32.08

*IC50* half-maximal inhibitory concentration, *CC50* half-maximal cytotoxic concentration, *SI* selectivity index.

**Fig. 1:**
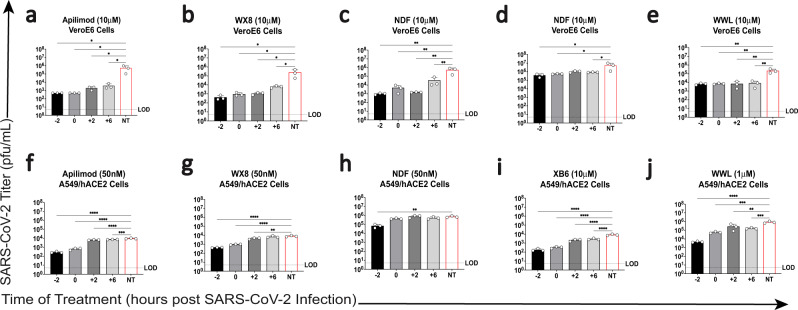
Time of addition analysis for PIKfyve inhibitor treatment in vitro. Time of addition analysis was performed by administering treatment either 2 h pre-infection, at the time of infection, or 2- or 6-h post-infection with SARS-CoV-2 in (**a**–**e**) VeroE6 cells or (**f**–**j**) A549/hACE2 cells. Supernatant titers at 24 h post infection are shown for **a**, **f** Apilimod, **b**, **g** WX8, **c**, **h** NDF, **d**, **i** XB6, and **e**, **j** WWL as compared to independent, no treatment controls. Ordinary one-way ANOVA analysis was used to compare differences in supernatant viral titer between treated cells and the no treatment control (red); *p ≤ 0.1, **p ≤ 0.01, ***p ≤ 0.001, ****p ≤ 0.0001. NT, no treatment; LOD: limit of detection.

Following these results, we aimed to ascertain which steps of the SARS-CoV-2 lifecycle PIKfyve inhibition may affect by performing time-of-addition analysis in the same cell lines. PIKfyve inhibitor treatment was initiated in triplicate at 2 h pre-infection, at the time of infection, or 2- or 6-h post-infection with SARS-CoV-2 (WA-1), and supernatants were collected after 24 h to assess virus output by plaque assay (Fig. 1). In addition, cellular RNA was collected to assess SARS-CoV-2 sgRNA levels as a measure of intracellular viral replication (Supplementary Fig. 2a). Compound efficacy at these timepoints would affect different aspects of the virus life cycle: efficacy exclusively at 2 h pre-infection or at the time of infection would suggest an effect on viral attachment/entry; efficacy at the earlier timepoints and at 2 h post-infection would suggest an effect on middle life cycle stages, such as viral transcription or translation; and compound efficacy at the previous timepoints as well as 6 h post-infection would suggest an effect on virion assembly or exit. Apilimod, WX8, and NDF showed steadily decreasing efficacy in VeroE6 cells as the treatment was administered progressively later during SARS-CoV-2 infection (Fig. 1a–c), suggesting that these inhibitors may be affecting multiple stages (e.g., entry and exit) of the virus lifecycle in this cell type. However, efficacy for these three compounds seemed to be limited to earlier timepoints when used in A549/hACE2 cells (Fig. 1f–h), suggesting the compounds may only affect entry in this cell type. Alternatively, WWL lacked this decreasing efficacy in VeroE6 cells (Fig. 1e), showing the same decrease is SARS-CoV-2 output titer at all treatment times, suggesting an effect on late stages of the viral life cycle. XB6 showed a decreased effect when administered later during infection for A549/hACE2 cells (Fig. 1i) and variable efficacy in VeroE6 cells (Fig. 1d).

### PIKfyve inhibitors exacerbate disease in a murine model of COVID-19 when administered pre- or post-infection

**Fig. 2:**
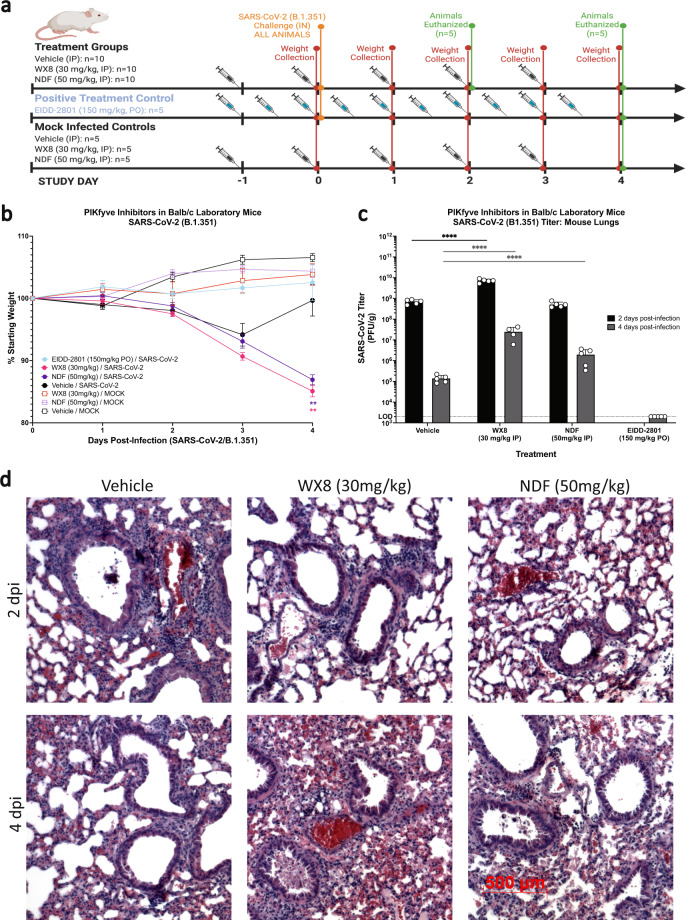
Pre-exposure prophylactic efficacy of PIKfyve inhibitor treatment against SARS-CoV-2 infection in mice. **a** Groups of mice were treated intraperitoneally with PIKfyve inhibitors WX8 or NDF once daily beginning 1 day pre-intranasal-challenge with 1 × 10^5^ plaque forming units SARS-CoV-2 (B.1.351). EIDD-2801 dosed twice a day was used as a positive treatment control and uninfected treatment controls were included to assess cytotoxicity. Image created using BioRender. **b** Weight changes were determined for 4 days post-challenge, plotted as the group mean with error bars indicating the ±SD. **c** Infectious viral loads from lung homogenates at 2 (black) or 4 (gray) days post SARS-CoV-2 challenge. **d** Lungs were collected at 2- or 4-days post-challenge and stained with hematoxylin and eosin to assess bronchiolar and alveolar damage and immune cell infiltration (500-μm scale bar shown at bottom left, representative for all panels). Mixed-effects analysis was used to compare differences in weight change or viral loads from lung homogenates between infection treatment groups and the vehicle-treated control group; **p ≤ 0.01, ****p ≤ 0.0001. dpi, days post-infection; PO, oral dosing; IP, intraperitoneal; IN, intranasal.

Two members of the WX8 family of PIKfyve inhibitors, WX8 and NDF, were tested for efficacy when treatment was initiated prior to infection with SARS-CoV-2 (B.1.351, “beta” variant, 1 × 10^5^ plaque forming units [PFU]/mL) in Balb/c laboratory mice (Fig. 2a). Apilimod was not available for pre-infection testing but was included for post-infection testing, described later. For this study, WX8 (30 mg/kg) and NDF (50 mg/kg) were dosed at the maximally tolerable concentration. Preliminary testing found that dosing at concentrations twice the dose caused lethality in mice. Treatment with WX8 or NDF resulted in increased weight loss as compared to vehicle-treated controls, showing a >10% weight loss by 4 days post-infection (dpi) for treated animals (Fig. 2b). This weight loss was absent for uninfected, treated controls. SARS-CoV-2 titers from lungs were also significantly higher in WX8-treated animals on 2 and 4 dpi and in NDF-treated animals on 4 dpi (Fig. 2c). Titer results were confirmed by RT-qPCR (Supplementary Fig. 2b). In contrast, treatment with the potent SARS-CoV-2 inhibitor EIDD-2801, used in the present study as a positive compound control, showed minimal weight loss and a complete reduction in SARS-CoV-2 titers in lungs^24,25^. Histopathological examination of lung sections from WX8-, NDF-, or vehicle-treated animals infected with SARS-CoV-2 showed epithelial sloughing in the bronchiolar space and inflammation in both the bronchiolar and alveolar spaces for vehicle-treated animals on 2 dpi. In contrast, inflammation was markedly reduced on 2 dpi for WX8- and NDF-treated animals (Fig. 2d). No qualitative differences between treatments could be discerned at 4 dpi.

**Fig. 3:**
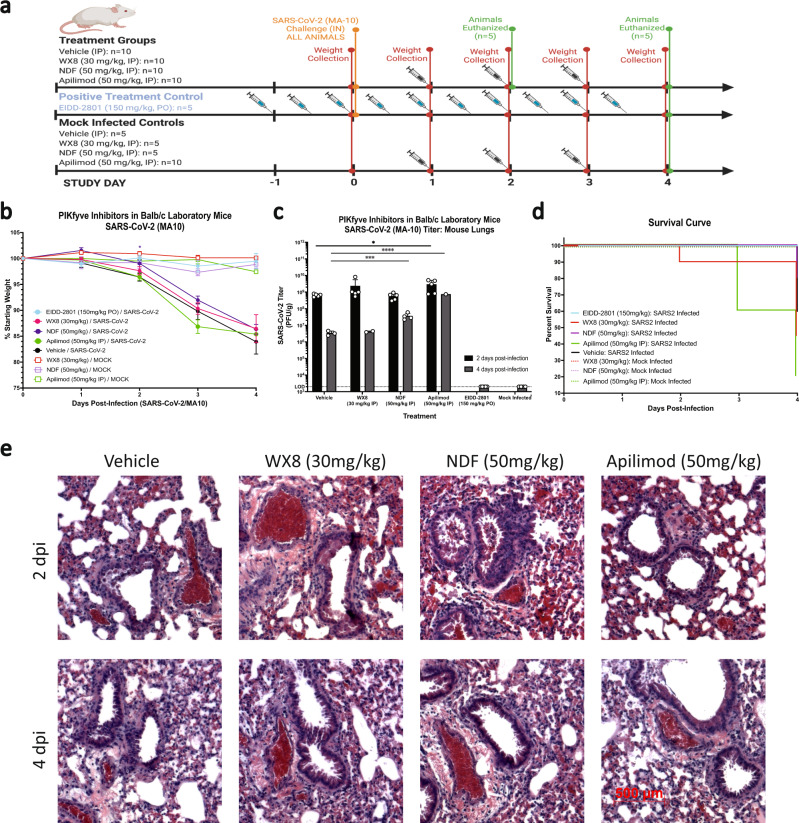
Post-exposure treatment efficacy of PIKfyve inhibitor treatment against SARS-CoV-2 infection in mice. **a** Groups of mice were treated intraperitoneally with PIKfyve inhibitors Apilimod, WX8, or NDF once daily beginning 1 day post-intranasal-challenge with 1 × 10^3^ plaque forming units SARS-CoV-2 (MA-10). EIDD-2801 dosed twice a day was used as a positive treatment control and uninfected treatment controls were included to assess cytotoxicity. Image created using Biorender. **b** Weight changes were determined for 4 days post-challenge, plotted as the group mean with error bars indicating the ±SD. **c** Infectious viral loads from lung homogenates at 2- (black) or 4- (gray) days post SARS-CoV-2 challenge. **d** Survival curves for treatment groups. **e** Lungs were collected at 2- or 4-days post-challenge and stained with hematoxylin and eosin to assess bronchiolar and alveolar damage and immune cell infiltration (500-μm scale bar shown at bottom left, representative for all panels). Mixed-effects analysis was used to compare differences in weight change or viral loads from lung homogenates between infection treatment groups and the vehicle-treated control group; *p ≤ 0.1, ***p ≤ 0.001, ****p ≤ 0.0001. dpi, days post-infection; PO, oral dosing; IP, intraperitoneal; IN, intranasal.

Following these results, Apilimod, WX8, and NDF were tested for efficacy against SARS-CoV-2 infection with treatment administration initiated 1 dpi (Fig. 3a). Given the anti-inflammatory results seen from histopathological examination of lung sections during pre-exposure testing, a more severe COVID-19 disease model (mouse-adapted SARS-CoV-2, MA-10, 1 × 10^5^ plaque forming units [PFU]/mL) was chosen for post-exposure efficacy testing. Apilimod dosing used was slightly higher than previously published data on efficacy testing against Ebola virus in mice^20^. None of the treatments tested were able to mitigate disease-associated weight loss (Fig. 3b), reduce SARS-CoV-2 titers from lung tissues (Fig. 3c, RT-qPCR in Supplemental Fig. 2c), or increase disease survival (Fig. 3d). In fact, viral clearance was delayed in NDF- and Apilimod-treated animals, and WX8- and Apilimod-treated animals died earlier than the vehicle control group. In addition, it was observed that PIKfyve inhibitor-treated mice developed what appeared to be conjunctivitis one day prior to succumbing to disease, which was absent in vehicle-treated animals that died or in uninfected, treated control. Histopathological analysis showed no discernible qualitative differences in inflammation as compared to vehicle-treated animals (Fig. 3e).

### PIKfyve inhibitors modulate immune parameters in vitro and in vivo

**Fig. 4:**
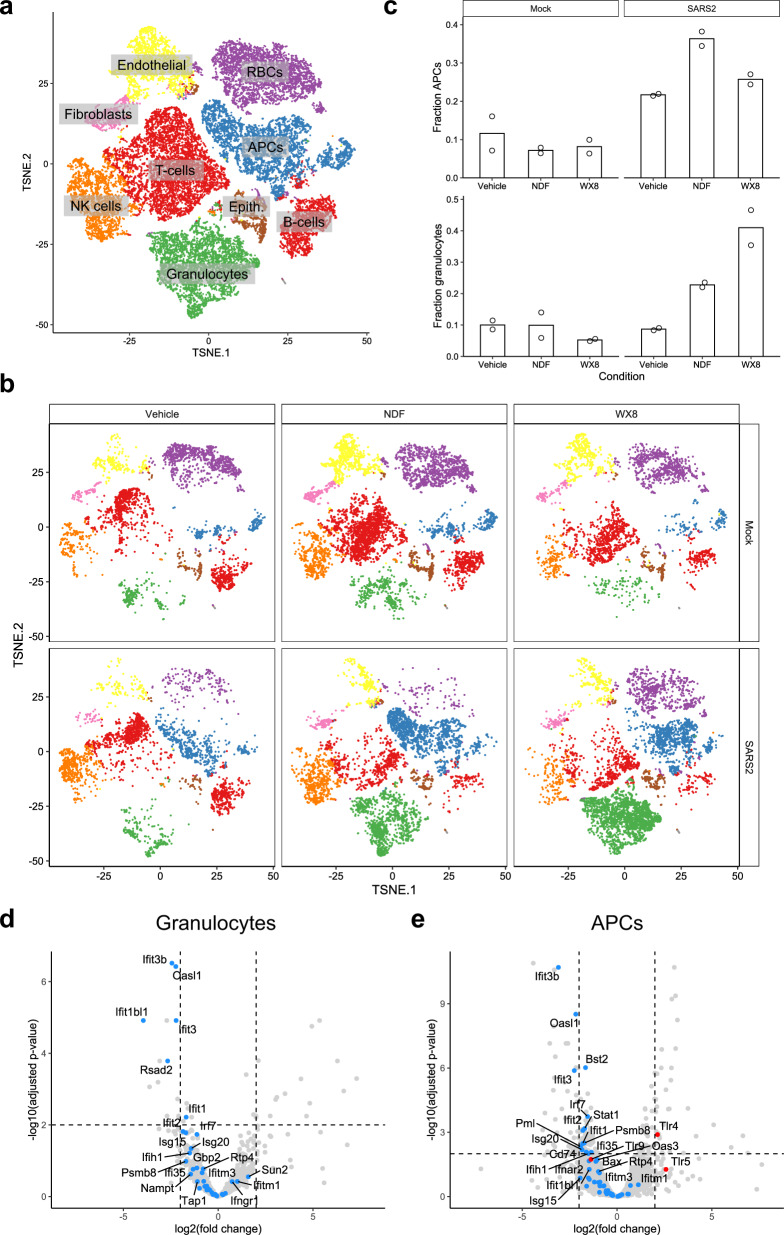
Single-cell sequencing analysis for PIKfyve inhibitor-treated animals against SARS-CoV-2 infection in mice. **a**, **b** t-SNE plot of scRNAseq data for **a** all samples or **b** faceted by condition. Cells are colored by cluster (k-means, *n* = 9), and clusters are labeled by cell type. **c** Bar plot showing the fraction of cells within the APC (top) and granulocyte (bottom) clusters. **d**, **e** Volcano plot showing differentially expressed genes in SARS-CoV-2-infected cells treated with WX8 versus untreated cells in the **d** granulocyte and **e** APC clusters. ISGs and genes in the interferon signaling pathway are colored blue (and labeled where space allows). TLRs in (**e**) are colored red. APC, antigen-presenting cells; Epith., epithelial cells.

Single-cell sequencing was performed on lungs collected on 4 dpi from the initial pre-infection treatment mouse study. Lung dissociation treatment was biased toward collection of immune cells so lung epithelial cells were underrepresented in our results. T-distributed stochastic neighbor embedding (t-SNE) analysis of single-cell expression data revealed 9 distinct cell clusters for each mouse group assessed (Fig. 4a). By 4 dpi, treatment of SARS-CoV-2-infected mice by either NDF or WX8 resulted in a marked increase in granulocytes and antigen-presenting cells as compared to vehicle-treated animals (Fig. 4b, quantified in Fig. 4c). In both the granulocyte (Fig. 4d, shown for WX8) and APC populations (Fig. 4e, shown for WX8), we identified reduced expression of interferon signaling pathway genes in SARS-CoV-2-infected lungs with treatment of either drug: NDF vs. untreated, granulocyte cluster (*z* = −2.2, *p* = 7.7 × 10^−5^); WX8 vs. untreated, granulocyte cluster (*z* = −2.6, *p* = 8.1 × 10^−7^); NDF vs. untreated, APC cluster (*z* = −1.0, *p* = 3.9 × 10^−2^); WX8 vs. untreated, APC cluster (*z* = −3.0, *p* = 2.3 × 10^−6^). Negative z-scores indicate predicted pathway inhibition^26^. In addition, Toll-like receptor (TLR) expression was significantly different in antigen-presenting cells, showing a down-regulation of TLR9 and an upregulation of both TLR4 and TLR5 (Fig. 4e, shown for WX8). In addition to these single-cell sequencing results, we also found that WX8, NDF, and Apilimod modulate secretion of proinflammatory cytokines in response to infection by another human coronavirus, hCoV-OC43, in studies with cultured HUVECs. Treatment with WX8, NDF, or Apilimod resulted in significant dose-dependent decreases in production of IFN-β, IL-1α, and IL-6, with a modest decrease in CXCL10 expression (Supplementary Fig. 1b–f).

## Discussion

The development of broad-spectrum antivirals continues to be a top priority to prepare for sudden viral disease outbreaks. PIKfyve inhibitors, including Apilimod, have shown to be potent antiviral agents in vitro against many pandemic- and epidemic- causing viruses (e.g., SARS-CoV-2, Ebola virus, Marburg virus)^17,23^. However, there remains a lack of published data on the effects of PIKfyve inhibitor treatment on disease progression in any animal models. In the present study, we similarly found that Apilimod and the WX8 family of PIKfyve effectively restrict SARS-CoV-2 replication in multiple cell lines, with activity observed down to nanomolar concentrations. Mechanistically, PIKfyve inhibitors appear to affect multiple stages of the virus lifecycle based on the in vitro time of addition analysis demonstrated here, though additional assays are still needed to confirm inhibition of specific lifecycle stages. The potential for other mechanisms of action later in the virus life cycle adds to the previously published data suggesting Apilimod blocks viral content release from endosomes^23^. However, though these and past in vitro results suggest that PIKfyve inhibitors have potential value as COVID-19 treatments, this work highlights that in vitro efficacy does not always translate to compound efficacy in vivo.

Animals treated with PIKfyve inhibitors generally experienced worse disease progression, regardless of when the treatment regimen was begun. Pre-exposure prophylactic administration resulted in an increase in peak viral load and delayed viral clearance from lungs. In addition, it was observed that trafficking of immune cells was delayed in the PIKfyve inhibitor-treated animals as compared to vehicle-treated animals by histology. These results were consistent with in vitro findings in human cells showing that PIKfyve inhibitors suppress secretion of proinflammatory cytokines (e.g., various interleukins) that normally promote recruitment and trafficking of immune cells. Together, these findings suggest a potential immune modulatory effect of PIKfyve inhibition, which in turn leads to delayed viral clearance. Though these results showed a worsening of disease, the observed immune modulatory effects of PIKfyve inhibitor treatment raised the possibility that a post-exposure treatment regimen could mitigate some of the effects of a cytokine storm, which has been correlated with COVID-19 disease severity^1,27^. Therefore, we decided to test this hypothesis using a more severe COVID-19 mouse model that utilized infection with a mouse-adapted SARS-CoV-2 (MA-10). However, we once again observed a similar worsening of disease using this therapeutic regimen and PIKfyve treated, SARS-CoV-2-infected animals died from disease earlier than vehicle-treated animals.

Single-cell analysis of immune cells from lungs collected during the pre-exposure treatment study suggests potential mechanisms for the increased death and lack of viral control in the lungs. PIKfyve treatment led to a marked increase of innate immune cells at 4 dpi, including neutrophils and antigen-presenting cells, as compared to untreated animals. Though there was a marked increase in these cells, the expression of interferon-stimulated genes in both cell groups was significantly decreased, suggesting a muted interferon response despite the increased viral load. Among the antigen-presenting cell cluster, Toll-like receptor (TLR) expression patterns suggested a response tuned more toward bacterial infection (higher TLR4 and TLR5 expression). In addition, a lower expression of TLR9 may suggest an inhibition of trafficking of plasmacytoid dendritic cells (pDC) to the lung, which have been suggested to control acute lung inflammation following lung damage^28^. Indeed, a reduction in pDCs could explain the lack of an interferon response in both APCs and granulocytes, as they are high producers of type 1 interferon. However, additional studies will need to be completed to assess these potential mechanisms.

This is the first published work to demonstrate that PIKfyve inhibitors are not protective in a mouse model of COVID-19 and led to increased disease. The compounds tested in this study showed a stark decrease on lung pathology and increase in lung SARS-CoV-2 titers, suggesting that the dosing regimens used provided enough bioavailability in the serum or lungs of these animals to have immune-modulatory effects. However, without pharmacokinetic data, it still remains possible that treatment with more bioavailable PIKfyve inhibitors may allow for the antiviral activity observed in vitro to have more of an effect in vivo, overriding the negative effects of immune-modulation. In addition, as with any animal disease model, the mouse system used in this study models many aspects of human infection and disease but does not necessarily fully recapitulate all aspects. Additional testing in other animal models (e.g., hamster, nonhuman primate) will need to be completed to confirm the findings from this study. Though these data suggest PIKfyve inhibitors may not be viable COVID-19 therapeutic, Apilimod remains a strong therapeutic candidate for other diseases, including positive human results against cancers and neurodegenerative disorders^14,16,18^. These animal model data will hopefully be informative for any future efficacy testing of this family of compounds as a disease treatment, including identifying additional pathways for antiviral targeting.

## Methods

### Virus and cells

All work with SARS-CoV-2 was performed in an A/BSL3 laboratory and approved by our Institutional Biosafety Committee (IBC# IBC-00005484) and Institutional Animal Care and Use Committee (IACUC# 1120004). Vero E6 cells (ATCC CRL 1586) were cultured in DMEM medium (Quality Biological) supplemented with 10% (vol/vol) heat-inactivated FBS (Sigma), 1% (vol/vol) penicillin–streptomycin (Gemini Bio-Products), and 1% (vol/vol) l-glutamine (2 mM final concentration; Gibco) (Vero medium). A549/hACE2 cells were generously provided by Dr. Brad Rosenberg and cultured in DMEM medium (Quality Biological) supplemented with 10% (vol/vol) heat-inactivated FBS (Sigma) and 1% (vol/vol) penicillin–streptomycin (Gemini Bio-Products)^29^. Human umbilical vein endothelial cells (HUVEC, Lonza C2519A) were maintained in EGM-2 media (Lonza CC-3162). HUVEC plates were coated with 0.1% gelatin for 30 min at 37 °C prior to seeding in black 96-well plates. All cells were maintained at 37 °C (5% CO_2_).

SARS-CoV-2 expressing GFP and the mouse-adapted SARS-CoV-2 (MA-10) was generously provided by Dr. Ralph Baric^30,31^. SARS-CoV-2 B.1.351 isolate was generously provided by Dr. Andy Pekosz. The original strain of SARS-CoV-2 was provided by the CDC following isolation from a patient in Washington State (WA-1; BEI number NR-52281). Stocks were prepared by infection of Vero E6 cells for 2 days when a cytopathic effect was starting to become visible. The media were collected and clarified by centrifugation before being aliquoted for storage at −80 °C. The titer of the stock was determined by plaque assay using Vero E6 cells (below).

### SARS-CoV-2 titer determination by plaque assay

Plaque assays were performed as described previously^32^. Briefly, 12-well plates were seeded with 2 × 10^5^ VeroE6 cells/well one day prior to processing. On the day of processing, media was removed from the 12-well plates and 200 μL of dilutions of lung homogenates or virus stock in DMEM were added to each well. Plates were incubated at 37 °C (5% CO_2_) for 1 h with rocking every 15 min. Following incubation, 2 mL of plaque assay media, DMEM containing 0.1% agarose (UltraPure™), and 2% FBS (Gibco), was added to each well and incubated for 3 days at 37 °C (5% CO_2_). Following incubation, plates were fixed with 4% paraformaldehyde, stained with 0.25% crystal violet (w/v), plaques counted, and titers calculated as plaque forming units (PFU).

### SARS-CoV-2 titer determination by RT-qPCR

RT-qPCR was processed for time of addition analysis (cellular RNA) and for mouse lung homogenates as described previously^32^. RNA was extracted per the manufacturer’s instructions using the Direct-zol RNA Miniprep Kit (Zymo Research). RNA was converted into cDNA (Thermo RevertAid Reverse Transcriptase) and used as template for qPCR (Qiagen RT2 SYBR green qPCR Mastermix). The primers used were against the N gene (5′-TAATCAGACAAGGAACTGATTA-3′ (Forward) and 5′-CGAAGGTGTGACTTCCATG-3′ (Reverse)) on an Applied Biosystems QuantStudio 5 thermocycler.

### In vitro compound efficacy testing

Efficacy testing was processed as described previously^33^. Briefly, infection and drug testing using GFP-expressing SARS-CoV-2 were performed in Vero E6 and A549/hACE2 cells. The cells were plated in clear-bottom black 96-well plates 1 day before infection. The cells were pretreated with drug at a range of concentrations for 2 h at 37 °C (5% CO_2_) before infection with SARS-CoV-2 (GFP) at MOI = 0.1. The plates were then incubated at 37 °C (5% CO_2_) for 48 h, followed by fixation with 4% paraformaldehyde, nuclear staining with Hoechst 33342 (Invitrogen), and data acquisition on a Celigo five-channel imaging cytometer (Nexcelom Bioscience). The percentage of infected cells was determined for each well based on GFP expression by manual gating using the Celigo software. In addition to plates that were infected, parallel plates were left uninfected to monitor the cytotoxicity of the drug alone. The plates were incubated at 37 °C (5% CO_2_) for 48 h before performing CellTiter-Glo (CTG) assays as per the manufacturer’s instructions (Promega). Luminescence was read on a BioTek Synergy HTX plate reader using the Gen5 software (v7.07; BioTek Instruments Inc.).

To measure the impact of selected drugs on HCoV-OC43 infection, 96-well plates seeded with Human Umbilical Vein Endothelial Cells (HUVEC) are infected with HCoV-OC43 and treated with the drugs. Uninfected or vehicle-treated cells (0.1% DMSO) are included as controls. HUVEC plates are predosed with drugs overnight at 34 °C at indicated concentrations (100 µl/well). The following day, HCoV-OC43 is added to the wells (100 µl/well) at an MOI of 0.2 and incubated for 3 h at 34 °C. After incubation, the medium containing virus and drugs is aspirated, the wells are washed with PBS+/+, new media is added with fresh drugs (100 µl/well), and the plates are incubated for 72 h at 34 °C. The supernatant is collected and stored for cytokine analysis and the plates are fixed with 4% Paraformaldehyde for Hoechst and/or antiviral staining. Fixed HUVECs are permeabilized with 100 µl/well of 0.1% Triton X, 1% FBS in PBS+/+ for 10 min at room temperature (RT), washed once with PBS, incubated in 100 µl/well of 1:50 human FcR Blocking Reagent (Miltenyi) in staining buffer (1% FBS in PBS+/+) for 30 min at RT, and washed again with PBS. Next, they are incubated with 100 µl/well of 2.5 μg/mL Anti-OC43 coronavirus primary antibody (EMD Millipore MAB9013) in staining buffer for 30 min at RT, washed five times with PBS, and then incubated with 100 µl/well of 1.6 μg/mL donkey anti-mouse HRP secondary antibody (Jackson ImmunoResearch 715-036-151) and 1:2000 Hoechst 33342 (Life Technologies H3570) in staining buffer for 1 h at RT. After five PBS washes, Hoechst fluorescence is read at 355 nm excitation and 450 nm emission using a Synergy H1 Spectrophotometer. Subsequently, viral load is measured using the ImmPACT DAB Peroxidase (HRP) Substrate kit (Vector Labs) according to the manufacturer’s instructions, and DAB absorbance was read at 465 nm.

### Compound time of addition studies

Time of addition analysis was performed in VeroE6 and in A549/hACE2 cells for all PIKfyve inhibitors available to us. Drug treatment was initiated either 2-h pre-infection (−2 h), at the time of infection (0 h), or 2- or 6-h post-infection (+2 h and +6 h, respectively) with SARS-CoV-2 (WA-1, MOI = 0.5), with virus added at 0 h and removed at the 2 h timepoint. Supernatants and cellular RNA in TRIzol (Ambion) were collected at 24-h post-infection. Supernatant was titered by plaque assay and cellular RNA in TRIzol was titered by RT-qPCR.

### Compound efficacy testing in a mouse model of COVID-19

Animal model testing was performed as described previously^24,34^ and approved by the University of Maryland, Baltimore Institutional Animal Care and Use Committee (IACUC# 1120004). Briefly, 8–10-week-old BALB/c laboratory mice were purchased from Charles River Laboratories (Wilmington, MA) and housed in the animal facility or in the ABSL3 laboratory at the University of Maryland, Baltimore. On the day of SARS-CoV-2 infection, mice were anaesthetized with a mix of xylazine and ketamine diluted in phosphate-buffered saline prior to intranasal inoculation with either 1 × 10^5^ PFU of SARS-CoV-2 B.1.351 or 1 × 10^3^ PFU of SARS-CoV-2 MA-10. Apilimod (50 mg/kg, Selleck Chemicals), WX8 (30 mg/kg, Specs, ChemDiv), and NDF (50 mg/kg, Specs, ChemDiv) treatments were administered once daily by intraperitoneal injection started either 1 day prior to infection (B.1.351) or 1 day following infection (MA-10). EIDD-2801 (150 mg/kg, WuXi AppTec) was dosed orally, twice daily, starting one day prior to infection was used as a positive control for all experiments, as it has been shown to be efficacious in animal models previously^24,25^. All compounds were formulated in corn oil (Sigma) with no more than 10% DMSO (Sigma). Weights were collected daily following infection and 5 mice sacrificed on 2 and 4 dpi for each treatment group. Lungs were harvested and sectioned for the following^1^: fixed in 4% paraformaldehyde for histopathological analysis^2^; homogenized in TRIzol for RT-qPCR analysis^3^; homogenized in PBS for plaque assay processing; and ref. ^4^ processed for single-cell sequencing (1 day prior to infection (B.1.351) experiment only, described below). Homogenization occurred using 1.0-mm glass beads (Sigma-Aldrich) and a Beadruptor (Omni International Inc.).

### Histopathology

Histopathology was processed as described previously^24,34^. Lung sections were fixed in 4% paraformaldehyde in phosphate-buffered saline for a minimum of 48 h, after which they were sent to the Histology Core at the University of Maryland, Baltimore, for paraffin embedding and sectioning. Five-micrometer sections were prepared and used for hematoxylin and eosin (H&E) staining by the Histology Core Services. Sections were imaged at ×10 magnification and assembled into figures using Adobe Photoshop and Illustrator software.

### Cytokine analysis in human umbilical vein endothelial cells

Cytokine analysis was conducted in multiplexed MSD plates using manufacturer provided calibrators and instructions (Mesoscale Discovery).

### Single-cell sequencing of mouse lungs

Mouse lung samples were collected from 2 mice per group on 4 dpi during the pre-infection-dosing, B.1.351 infection experiment. Lung samples were dissociated into a single-cell suspension using the gentleMACS Dissociator as described previously^35^. Single-cell suspensions were then processed using a 10X Chromium Controller to isolate single cells and cDNA/library preparation was completed using the Chromium Single Cell 5′ GEM preparation and i7 Multiplex Kit, according to the manufacturer’s instructions, followed by sequencing on a NextSeq Sequencer (Illumina).

scRNAseq data were processed and aggregated by the CellRanger pipeline (10X Genomics; software version 6.1.2) using the five-prime chemistry mode^36^. The 2020-A Mus musculus reference was used for transcript mapping (genome: GRCm38; annotation version: Gencode M23). After k-means clustering (k = 9), cell types were predicted using scmap (version: 1.14.0)^37^ and confirmed by inspecting differential expression patterns in the Loupe Browser (10X Genomics; software version 6.0.0) in R version 4.1.1^38^. Cells from the RBC cluster were removed from cluster-level analysis as differences in the number of RBCs are likely due to incomplete perfusion. Cluster-level differential expression analysis was performed using DEseq2 (version: 1.32.0), after an additional filtering step to remove RBCs–cells with detectable hemoglobin Hbb-a2 transcript (ENSMUSG00000069917) with five or more reads were removed. Pathway analysis was performed using Ingenuity Pathway Analysis (Qiagen Inc.)^26^. The raw reads have been deposited in the NCBI Sequence Read Archive (Accession Number PRJNA841980).

### Statistics and reproducibility

All statistics were performed using GraphPad PRISM (GraphPad Software, La Jolla, CA). Further information on research design is available in the Nature Research Reporting Summary linked to this article.

### Reporting summary

Further information on research design is available in the Nature Research Reporting Summary linked to this article.

## Supplementary information

Supplementary Information

Reporting Summary

## Data Availability

Single-cell sequencing data (raw reads) have been deposited in the NCBI Sequence Read Archive under accession number PRJNA841980 (used for Fig. 4).
