# Supplementary material for: PIKfyve-specific inhibitors restrict replication of multiple coronaviruses in vitro but not in a murine model of COVID-19
Source: Commun Biol. 2022 Aug 12;5:808. doi: 10.1038/s42003-022-03766-2 (PMC9372968; doi:10.1038/s42003-022-03766-2)
Supplement: Supplementary file 1 — Supplementary Information [file 42003_2022_3766_MOESM1_ESM.pdf]

Supplementary Figures for:

PIKfyve-specific inhibitors restrict replication of multiple human coronaviruses *in vitro* but not in a murine model of COVID-19

James Logue[1,2], Arup R. Chakraborty[3], Robert Johnson[1,2], Girija Goyal[4], Louis J. Taylor[1,2], Lauren Baracco[1,2], Marisa E. McGrath[1,2], Robert Haupt[1,2], Melissa Rodas[4], Brooke A. Furlong[4], Mercy Soong[4], Pranav Prabhala[4], Viktor Horvath[4], Kenneth E. Carlson[4], Stuart Weston[1,2], Donald E. Ingber[4-6], Melvin L. DePamphilis[3], Matthew B. Frieman[1,2,*]

[1]Department of Microbiology and Immunology, University of Maryland, School of Medicine, 685 West Baltimore St, Baltimore, MD, 21201, USA.

[2]Center for Pathogen Research, University of Maryland, School of Medicine, 685 West Baltimore St, Baltimore, MD, 21201, USA.

[3]Division of Developmental Biology, National Institute of Child Health & Human Development, National Institutes of Health, Bethesda, MD 20892-2790, USA.

[4]Wyss Institute for Biologically Inspired Engineering, Harvard University, Boston, MA 02115, USA.

[5]Harvard John A. Paulson School of Engineering and Applied Sciences, Cambridge, MA 02139, USA.

[6]Vascular Biology Program and Department of Surgery, Boston Children's Hospital and Harvard Medical School, Boston, MA 02115, USA.

[*]Correspondence: M.B.F. (mfrieman@som.umaryland.edu)

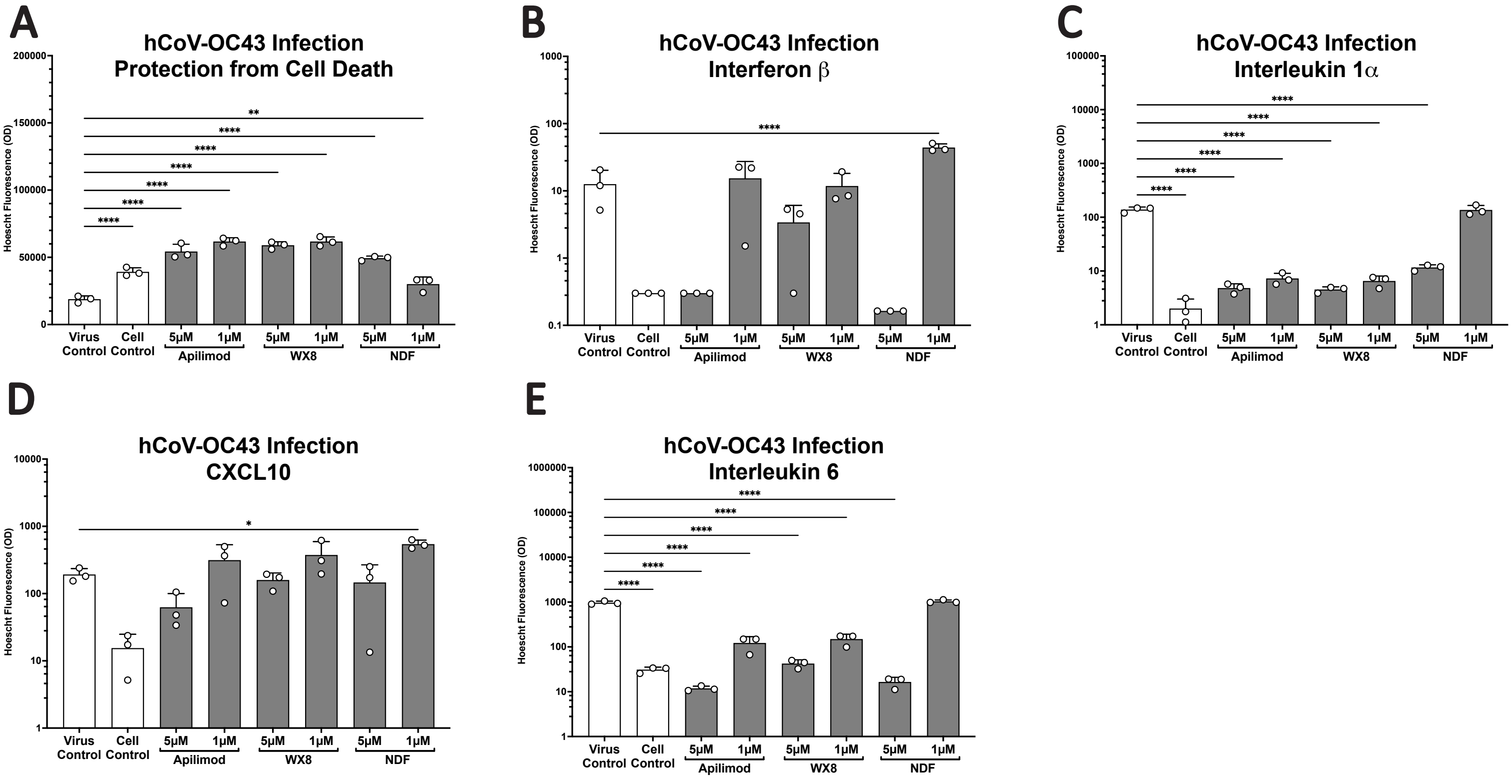

Supplementary Figure 1: Treatment efficacy of PIKfyve inhibitors against hCoV-OC43 infection *in vitro*. HUVECs were treated with PIKfyve inhibitors followed by infection with hCoV-OC43. (a) Efficacy of PIKfyve inhibitors against hCoV-OC43 infection-induced cell death. (b-f) Cytokine measurements from PIKfyve inhibitor treated cells infected with hCoV-OC43 for: (b) interferon beta; (c) interleukin 1 alpha, (d) CXCL10; and (e) interleukin 6. Ordinary one-way ANOVA was used to compare differences in cytokine production for each treatment group and uninfected control as compared to the untreated virus control; *$p \leq 0.1$, **$p \leq 0.01$, ****$p \leq 0.0001$.

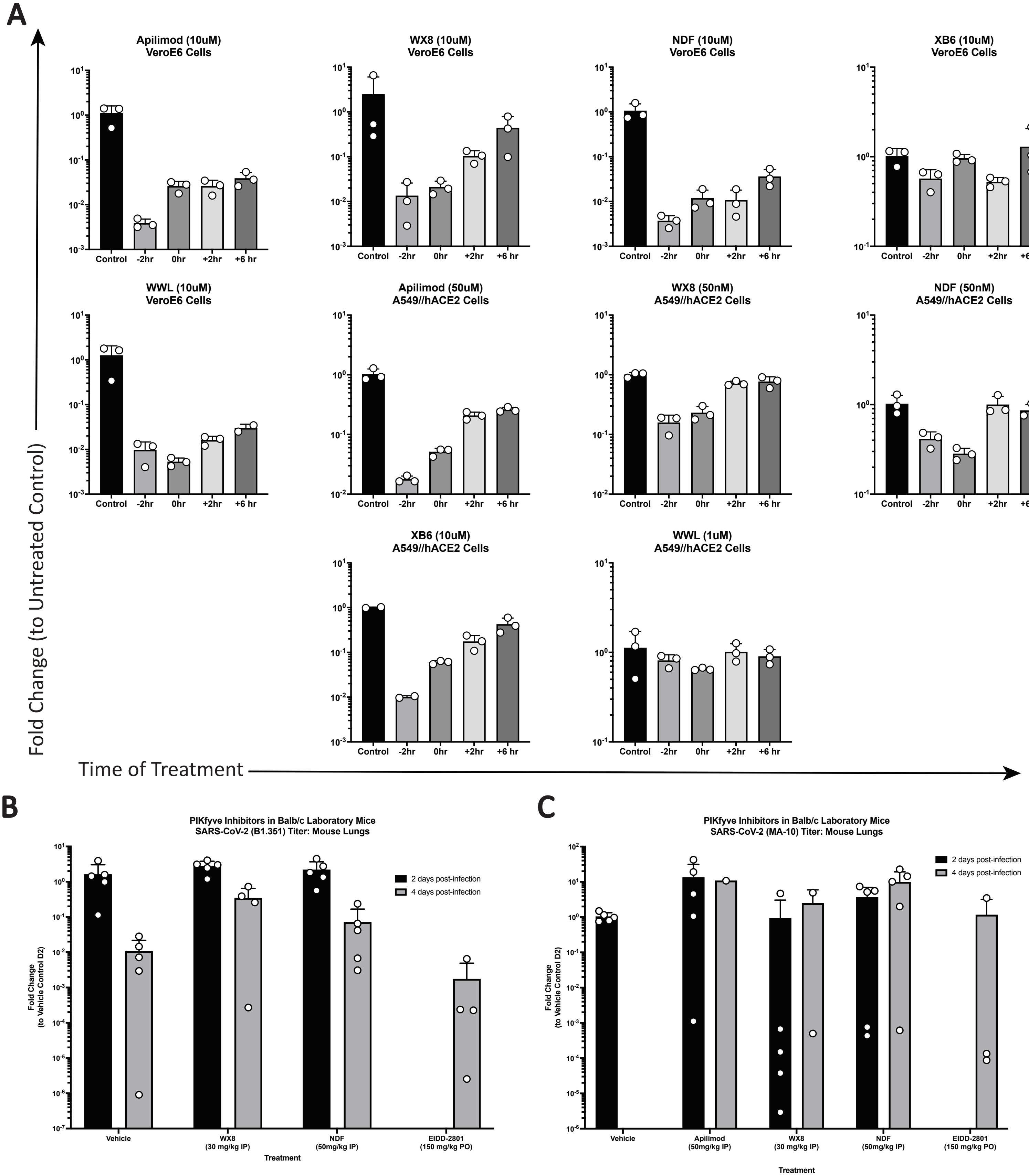

Supplemental Figure 2: SARS-CoV-2 sub-genomic RT-qPCR analysis of time of addition and mouse infection studies. RT-qPCR for sub-genomic SARS-CoV-2 RNA (replicative intermediate) was performed for (a) time of addition analysis in PIKfyve inhibitor treated VeroE6 or A549/hACE2 cells infected with SARS-CoV-2 or (b-c) to assess viral loads from lung homogenates at 2 (black) or 4 (gray) days post SARS-CoV-2 challenge for (b) pre-infection dosing initiation or (c) post-infection prophylactic dosing initiation. Ordinary one-way ANOVA was used to compare differences in sub-genomic RNA production for each treatment timepoint as compared to the NT control; *p ≤ 0.1, **p ≤ 0.01, ****p ≤ 0.0001. Abbreviations: "NT", no treatment; "sgRNA", subgenomic RNA.
